# The Association of Adropin with Asymptomatic Coronary Calcification in Patients in Early Stages of Chronic Kidney Disease

**DOI:** 10.3390/ijms26167816

**Published:** 2025-08-13

**Authors:** Tetiana A. Berezina, Oleksandr O. Berezin, Evgen V. Novikov, Alexander E. Berezin

**Affiliations:** 1 Department of Internal Medicine and Nephrology, VitaCenter, 69000 Zaporozhye, Ukraine; talexberezina@gmail.com; 2 Luzerner Psychiatrie AG, 4915 St. Urban, Switzerland; lunik.mender@gmail.com; 3 Department of Functional Diagnostics, Shupyk National Healthcare University of Ukraine, 04136 Kyiv, Ukraine; doctornovikov@ukr.net; 4 Department of Internal Medicine II, Division of Cardiology, Paracelsus Medical University, 5020 Salzburg, Austria

**Keywords:** chronic kidney disease, vascular calcification, adropin, circulating biomarkers, prediction

## Abstract

Early stages of chronic kidney disease (CKD) are closely associated with vascular remodeling and coronary artery calcification. The aim of this study is to determine whether adropin is associated with asymptomatic coronary calcification in patients in the early stages of CKD. This study enrolled 337 individuals fulfilling the inclusion criteria of the early stages of CKD (G1–2, A1–3) and divided them into two subgroups with (*n* = 196) and without (*n* = 141) asymptomatic coronary artery calcification. Native coronary multi-detector computed tomography angiography was conducted to determine coronary artery calcification, which was stratified into four grades according to the Agatston method. Serum levels of adropin were measured by ELISA. The patients with known asymptomatic coronary artery calcification had significantly lower levels of adropin than those without this condition. The levels of adropin in individuals with mild (130–199 HU), moderate (200–299 HU), severe (300–399 HU) and very severe (≥400 HU) calcification were 3.13 (95% CI = 1.92–4.21) ng/mL, 2.3 (95% CI = 1.45–3.6) ng/mL, 2.1 (95% CI = 1.22–3.25) ng/mL and 1.26 (95% CI = 1.13–1.98) ng/mL, respectively. In multivariate logistic regression low adropin (<2.95 ng/mL), a presence of hypertension, type 2 diabetes mellitus (T2DM) exerted their independent potencies to predict asymptomatic coronary calcification. Moreover, adropin demonstrated better discriminative potency than concomitant hypertension and T2DM. Conclusions: Low levels of circulating adropin significantly predicted a risk of coronary artery calcification in patients in the early stages of CKD.

## 1. Introduction

Chronic kidney disease (CKD) is a leading cause of cardiovascular (CV) morbidity and mortality worldwide [1]. The estimated global prevalence of pre-dialysis CKD is 13.4% (11.7–15.1%), and the number of patients with end-stage kidney disease (ESRD) requiring renal replacement therapy is estimated to be between 4.902 and 7.083 million [1,2]. CKD is mainly driven by the increasing prevalence of diabetes mellitus, hypertension, obesity, dyslipidemia and aging [3,4,5].

CKD, especially at stage (G) 4–5, is markedly associated with increased CV risk manifested by coronary artery disease (CAD), heart failure, arrhythmias and sudden cardiac death [6]. In fact, in the early stages (G1–2), a chronic pro-inflammatory state along with attributable conventional CV risk factors contributes to myocardial and vascular remodeling processes leading to ectopic calcification, including cardiac valves and vascular calcification, as well as accelerating atherosclerosis and plaque development [6,7]. On the contrary, in CKD G 3–5, non-classical risk factors such as anemia, electrolyte disturbances including hyperphosphatemia, systemic and microvascular inflammation, oxidative stress, iso- and non-osmotic sodium retention, fluid overload and volume expansion, malnutrition/cachexia, sympathetic hyperactivity, osteoporosis, accumulation of “uremic toxins” and various hormonal disorders are the main factors influencing the prognosis [8]. Although an association of vascular remodeling with CV mortality has been found to be positively associated with CKD stage, coronary calcification is significantly associated with CKD-related CV events across all stages of CKD [9,10].

The Agatston method is the conventionally used system for quantifying the coronary artery calcium score [11]. Although the coronary artery calcium score is significantly associated with the occurrence of major CV events (MACEs), patients should be categorized as at-risk according to calcium accumulation that is defined as a density of coronary lesions above 130 Hounsfield units (HU) with the areas of hyperattenuation of at least 1 mm^2^ [11]. In this context, a certain number of asymptomatic patients with low-to-intermediate accumulation of calcium are likely to be underdiagnosed at the risk of MACEs, because this approach is particularly sensitive to the calcified plaques with high attenuation [12]. In addition, the Expert Consensus Document by the American College of Cardiology Foundation and the American Heart Association now recommends screening individuals at intermediate risk but did not find enough evidence to recommend coronary artery calcium testing and further stratification of those in the low- or high-risk categories for CAD [13]. Meanwhile, certain circulating biomarkers, such as matrix Gla protein, fetuin A, fibroblast growth factor 23, calciprotein particles and bone-related proteins (osteoprotegerin, osteopontin, sclerostin), have been suggested for prediction of plaque shaping and atherosclerosis severity in patients with CKD G3–5, but not in those with CKD G1–2 [14,15,16].

Recently, adropin, a newly-identified multifunctional secreted peptide encoded by the energy homeostasis-associated (Enho) gene, has been investigated for its potential role in the prediction of acute kidney disease-to-CKD transition and monitoring CKD progression [17,18,19]. Adropin is involved in the regulation of energy homeostasis, vasodilation via the expression of endothelial nitric oxide synthase, insulin sensitivity through promoting insulin signaling pathways (Akt phosphorylation and the activation of the glucose transporter 4 receptor), reducing endogenous hepatic gluconeogenesis, lipids oxidation and enhancing glucose utilization [20]. There is evidence that adropin, acting through the phosphatidylinositol 3-kinase (PI3K)/protein kinase B (Akt) /mTOR signaling pathway, potentially protects against inflammation, oxidative damage, accelerating atherosclerosis and plaque formation [21]. Previous clinical studies have shown that low serum adropin levels were associated with severity of coronary atherosclerosis, as reflected by higher SYNTAX score and Gensini score, either in patients undergoing percutaneous coronary intervention or in individuals without CKD [22,23,24]. Along with it, adropin is likely be a promising biomarker for predicting the onset of CAD in CKD individuals [25]. However, there is currently no specific evidence for the association between circulating levels of adropin and coronary calcification and plaque progression at early stages of CKD. The purpose of this study is to determine whether adropin is associated with asymptomatic coronary calcification in patients in the early stages of CKD.

## 2. Results

### 2.1. Baseline Clinical Characteristics

The entire study cohort comprised 337 patients (64.1% male; mean age: 65 years) with mean duration of CKD of 1.1 (0.4–1.8) years. Baseline characteristics were summarized in Table 1. Patients with asymptomatic coronary calcification were older and had higher prevalence in hypertension and type 2 diabetes mellitus, as well as higher levels of high-sensitivity C-reactive protein (hs-CRP), tumor necrosis factor-alpha (TNF-alpha) and lower levels of adropin than those without the condition.

No significant differences between subgroups were observed in sex, body mass index (BMI), waist circumference, waist-to-hip ratio, smoking, dyslipidemia, abdominal obesity, left ventricular hypertrophy (LVH), heart failure with preserved ejection fraction (HFpEF), systolic and diastolic blood pressure, hemodynamic parameters, eGFR, lipid profile, urinary albumin/creatinine ratio (UACR), serum uric acid, creatinine, glucose, calcium and phosphorus levels, Lp(a), soluble suppression of tumorigenicity-2 (sST2), interleukin (IL)-6, fetuin-A, fibroblast growth factor (FGF)-23. Additionally, the individuals with symptomatic coronary calcification were more frequently treated with angiotensin-II receptor blockers and calcium channel blockers with than those without coronary artery calcification. The mean duration of statin use was 2.5 (0.6–3.9) years and the patients from both subgroups did not differ in the duration of statin utilization (2.3 (0.4–4.3) years vs. 2.6 (0.6–3.5) years, respectively, *p* = 0.648). In contrast, in the cohort of non-coronary artery calcification ACE inhibitors were prescribed significantly more often than in another group.

### 2.2. Spearman’s Correlations Between the Levels of Circulating Biomarkers and Other Parameters in CKD G1–2 Patients with Asymptomatic Coronary Artery Calcification

Spearman’s correlations coefficients between the levels of circulating biomarkers and other parameters in CKD G1–2 patients with asymptomatic coronary artery calcification are shown in Table 2. The adropin levels were positively associated with global longitudinal strain (GLS), LV ejection fraction (LVEF) and Agatston density range and negatively correlated with the age, BMI, systolic and diastolic blood pressure, LV myocardial mass index (LVMMI), left atrial volume index (LAVI), UACR, fasting plasma glucose, total cholesterol, and LDL-cholesterol. The levels of hs-CRP were inversely correlated with LVMMI, whereas TNF-alpha exhibited borderline positive correlation with BMI.

### 2.3. The Levels of Adropin Depending on the Weighted Sum of Coronary Artery Lesions with a Density

**Figure 1:**
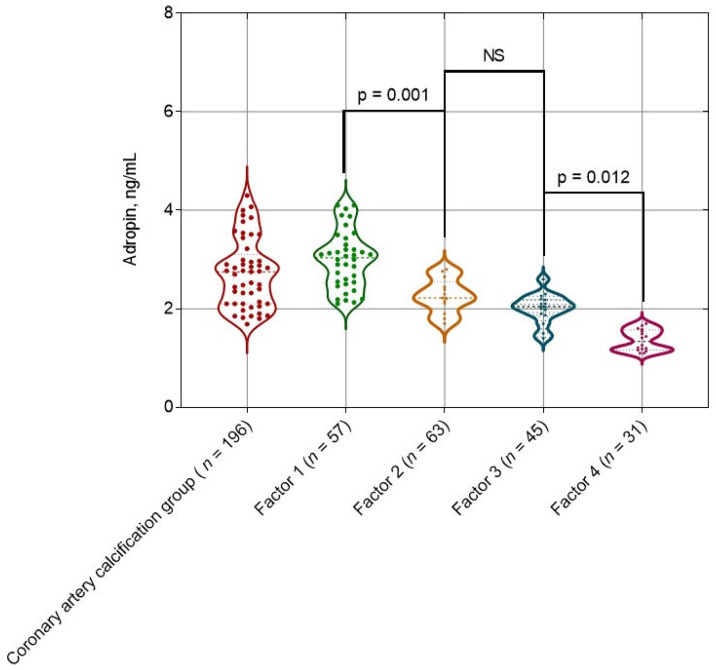
Violin plot of adropin concentrations in individuals with asymptomatic coronary calcification according to Agatston criteria. Notes: factor 1 = 130–199 HU, factor 2 = 200–299 HU, factor 3 = 300–399 HU and factor 4 = ≥ 400 HU. Abbreviations: NS, not significant, HU, Hounsfield units.

The levels of adropin in individuals with mild (130–199 HU), moderate (200–299 HU), severe (300–399 HU) and very severe (≥400 HU) calcification were 3.13 (95% CI = 1.92–4.21) ng/mL, 2.3 (95% CI = 1.45–3.6) ng/mL, 2.1 (95% CI = 1.22–3.25) ng/mL and 1.26 (95% CI = 1.13–1.98) ng/mL, respectively. The levels of adropin in individuals with different stages of coronary artery lesion showed the significant differences between mild and moderate calcification as well as between severe and very severe calcification (Figure 1).

### 2.4. Receiver Operating Characteristic Curve Analysis for Adropin

**Figure 2:**
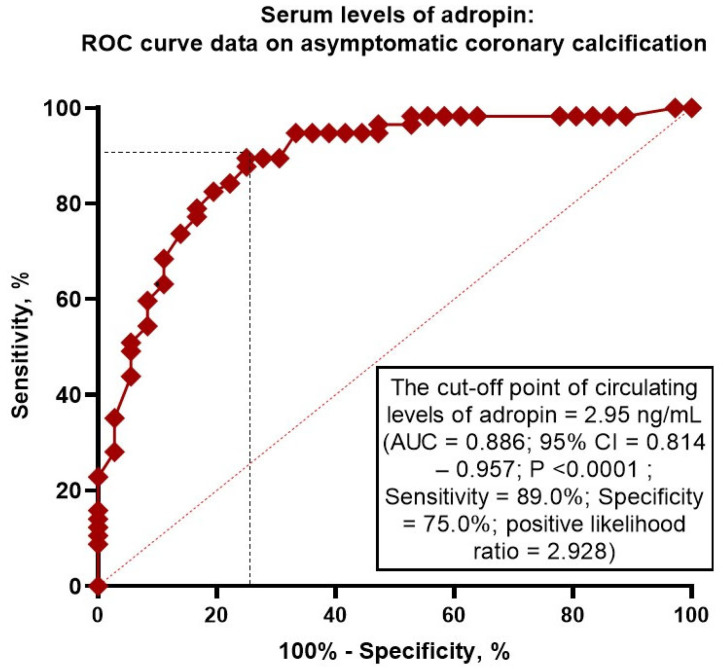
Receiver Operating Characteristic Curve Analysis for adropin. Abbreviations: AUC, area under curve; CI, confidence interval.

ROC curve analysis was performed to determine the optimal cut-off for adropin as possible predictor of asymptomatic coronary calcification (Figure 2).

We identified that the levels of adropin < 2.95 ng/mL (AUC= 0.886; 95% CI = 0.814–0.957; *p* < 0.0001; Sensitivity = 89%, Specificity = 75%, positive likelihood ratio = 2.928).

### 2.5. Predictors of Asymptomatic Coronary Calcification: Univariate and Multivariate Logistic Regression Analyses

We used the median serum levels of UACR (49 mg/g), hs-CRP (5.15 mg/L) and TNF-α (2.61 pg/mL) as cut-off points for further univariate and multivariate logistic regression analysis (Table 3). In a univariate logistic regression, low adropin (<2.95 ng/mL), higher levels of hs-CRP (≥5.15 mg/L) and TNF-alpha (≥2.61 pg/mL), as well as a presence of hypertension, T2DM, and HFpEF were found as positive predictors for asymptomatic coronary calcification. Additionally, administration of calcium channel blockers and SGLT2 inhibitors negatively predicted this condition. In multivariate logistic regression only the low adropin (<2.95 ng/mL), a presence of hypertension, and T2DM exerted their independent potencies to predict asymptomatic coronary calcification in patients in the early stages of CKD.

### 2.6. Comparison of the Predictive Models

We compared the predictive models for asymptomatic coronary calcification (Table 4) and found that Model 1 (low levels of adropin [<2.95 ng/mL]) was significantly (*p* < 0.001 for both cases) better than two other Models. Moreover, Model 2 (the presence of hypertension) and Model 3 (the presence of T2DM) did not differ from each other in their ability to predict asymptomatic coronary calcification.

## 3. Discussion

As cardiovascular complications are one of the factors that predispose patients to mortality in the early stages of CKD, simple and sensitive in vitro tests with high reproducibility are likely to be promising in stratifying patients at a higher risk of vascular remodeling and coronary artery calcification. Although significant cardiovascular risk is exacerbated by inflammation, hyperparathyroidism and the regulation of bone molecules involved in the progression of calcification, these pathogenetic factors intervene in plaque formation at the G3–5 stages of CKD [26]. On the other hand, it remains unclear whether full control for concomitant comorbidities, such as T2DM, obesity, osteoporosis, hypertension, provides sufficient benefits in the prevention of asymptomatic coronary artery calcification and whether these factors continue to be evident predictors for unfavorable clinical outcomes across all stages of CKD, but not at G3–5 stages [27]. In our multicenter study we found that low serum levels of adropin independently predicted asymptomatic coronary artery calcification in adequately treated patients in the early stages of CKD. Moreover, we also detected an inverse association of adropin concentrations with Agatston scores. Additionally, discriminative value of low levels of adropin was sufficiently better in comparison with the presence of conventional cardiovascular comorbidity, such as T2DM and hypertension.

Previous clinical studies have revealed than numerous biomarkers, such as bone-related proteins, brain-derived neurotrophic factor, FGF-23, IL-6, Klotho, Matrix Gla protein, were strongly associated with severe coronary artery calcification in patients with diabetes or hypertension at risk of CKD or without CKD [28,29,30]. Nevertheless, the biomarkers of inflammation (IL-6, TNF-alpha, hs-CRP, sST2, fetuin-A), kidney damage (cystatin C, UACR), calcium/phosphate metabolism (calcium, phosphate/pyrophosphate, parathyroid hormone, vitamin D), ectopic calcium accumulation (osteopontin, osteocalcin, Matrix Gla protein) have been exerted as indicators of calcified atherosclerotic plaque with clinically significant stenosis (>50%) and clinical signs and symptoms [31]. Overall, the patients in the early stages of CKD, even without clinical features of atherosclerotic cardiovascular disease (ASCVD), demonstrate life-threatening outcomes frequently associated with vulnerable plaque without serious calcium accumulation and hard plaque cap, while non-stenotic lesions of the coronary artery remain poorly diagnosed with conventional methods of native coronary visualization due to technical causes. Aligning with this, alternative approaches, including measurement of increased arterial wave velocity, the ankle–brachial index, as well as a diagnosis of diabetes and hypertension at CKD G1–2, appear to be approximation-based strategies rather than accurate risk assessments. This knowledge gap requires new approaches to stratify these at-risk individuals.

Adropin—a novel pleotropic peptide with organ-protective capacity—is involved in the pathophysiological mechanisms of CKD across all stages [32]. Despite adropin being initially recognized as a hepatokine, which modulates the responses of liver and muscle to insulin and glucagon, its expression was found in other organs and tissue including kidney parenchyma, vasculature, brain and myocardium [33]. Adropin contributes to energy homeostasis, glucose and lipids metabolism and mediates cardiovascular protection via enhancement of nitric oxide production, mediating vascular integrity and vasodilation, blood pressure regulation, prevention of ischemia kidney injury and atherosclerotic plaque formation [34]. Previous studies showed that adropin acts through several signaling pathways, i.e., the Nb-3/Notch, PI3K/Akt/mTOR, G-protein coupled receptor 19/mitogen-activated protein kinase/extracellular signal-regulated kinase 1/2, vascular endothelial growth factor receptor 2, and exerts anti-inflammatory, anti-apoptotic, angiopoietic, anti-oxidative and anti-proliferative properties [35].

Decreased serum levels of adropin were found in individuals in the end stage of renal disease (ESRD) [36,37]. Meanwhile, T2DM patients with CKD G3–5 had higher adropin levels than those with early CKD, whereas hypertensive individuals with advanced CKD, in contrast, had lower levels of adropin than those in the early stages of CKD [38,39]. In our study, the levels of adropin among patients with early CKD with known coronary calcification were lower than in individuals without vascular lesion with calcium accumulation.

As adropin probably plays one of the key roles in preventing the emergence of vulnerable atheroma, we hypothesized that its deficiency may contribute to vascular disintegrity and early calcification through several pathogenetic mechanisms, i.e., direct mediation of subintimal lipid oxidation, proliferation of vascular smooth muscle cells, macrophage transformation/migration, toll-like receptor activity and worsening of endothelial cell precursor activity. To the best of our knowledge, low levels of adropin may be detected in individuals with CKD before secondary electrolyte and metabolic disorders that are suitable for advanced stages of the disease [40]. Thus, the negative association of the circulating levels of adropin with Agatston stages of coronary calcification, which we first detected in the study, opens new perspectives for early screening of individuals who are either angiographically negative or have no clinically significant coronary artery stenosis. Another benefit of the findings is likely to be linked with the possibility of identifying asymptomatic patients at higher risk of coronary artery calcification beyond eGFR ≤60 mL/min/1.73 m^2^. Perhaps this could assist in increasing the sensitivity of native coronary multi-detector computed tomography angiography in this issue.

It is worth noting that the discriminatory potential of adropin not only exceeded that of comorbid conditions such as hypertension and diabetes but was found in optimally treated CKD G1–2 patients beyond the levels of kidney damage biomarkers. Although biomarkers of renal damage, such as IL-6, FGF-23 and sST2, have previously been associated with coronary atherosclerosis [18], they were not found to be superior to adropin in patients with early-stage CKD in the present study. Interesting, the majority of patients received RAAS inhibitors, and the proportion of the patients given SGLT2 inhibitors and CCB was approaching 20%. However, this is the first study to clearly establish the benefits of adropin as a biomarker of asymptomatic coronary artery calcification in optimally treated patients, according to current guidelines.

However, this study had several limitations. Firstly, we did not investigate whether patients’ nutritional status was related to their adropin concentration. Our hypothesis was based on the assumption that this would probably not provide significant additional information for patients in the early stages of CKD. Secondly, the lack of analysis of coronary atherosclerosis severity using traditional scales, such as the Gensini or GRACE scales, probably did not affect the results of the study. A final limitation relates to the relatively small patient sample, although the multicentrer design of the study overcomes the risks of statistical bias. Expanding the research to a larger patient population is likely to be necessary to ensure the method’s predictability and to evaluate the economic burden. Overall, we believe that these limitations will not affect the interpretation of our study results.

## 4. Materials and Methods

### 4.1. Study Population

**Figure 3:**
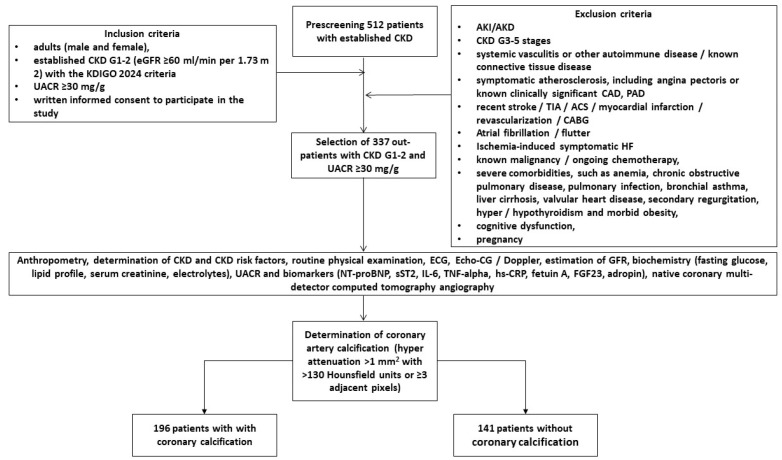
Flow chart and study design. Abbreviations: AKI, acute kidney injury; AKD, acute kidney disease; CKD, chronic kidney disease; CAD, coronary artery disease; CABG, coronary artery bypass grafting; ECG, electrocardiogram; HF, heart failure; UACR, urine albumin-to-creatinine ratio; eGFR, estimated glomerular filtration rate; NT-proBNP, N-terminal natriuretic pro-peptide; IL, interleukin; FGF, fibroblast growth factor; sST2, soluble suppression of tumorigenicity-2; TNF, tumor necrosis factor; hs-CRP, high-sensitive C-reactive protein.

We selected 512 white adults (male and female), who had established diagnoses of CKD, among whom 337 individuals fulfilling the inclusion criteria of the early stages of CKD (G1–2, A1–3) were consecutively enrolled from August 2022 to April 2025. All individuals were longitudinally evaluated at three centers: the private hospital “Vita Center” (Zaporozhye, Ukraine), the private medical center “Elitmedservice” (Zaporozhye, Ukraine) and the private hospital “MIRUM clinic” (Kyiv, Ukraine). The inclusion and exclusion criteria, as well as study procedures and determination of vascular calcification, are outlined in Figure 3. Inclusion criteria were both genders, age > 18 years, established early CKD (G1–2) with a urine albumin-to-creatinine ratio (UACR) ≥ 30 mg/g and written informed consent to participate in the study. Individuals with acute kidney injury (AKI), acute kidney disease (AKD), systemic vasculitis, autoimmune and connective tissue disease, a history of a symptomatic cardiovascular events and current symptomatic atherosclerotic cardiovascular disease, recent transient ischemic attack/stroke, acute coronary syndrome, myocardial infarction, previous revascularization/coronary artery bypass grafting, peripheral artery disease, ischemia-induced heart failure, known malignancy/ongoing chemotherapy, severe comorbidities (anemia, chronic obstructive pulmonary disease, pulmonary infection, bronchial asthma, liver cirrhosis, valvular heart disease, secondary regurgitation, hyper/hypothyroidism and morbid obesity), cognitive dysfunction and pregnancy were not enrolled in the study. All enrolled individuals were divided into two subgroups with (*n* = 196) and without (*n* = 141) asymptomatic coronary artery calcification.

### 4.2. Determination of Early Stages of CKD

CKD was defined, according to the 2024 Kidney Disease: Improving Global Outcomes CKD Work Group, as abnormalities in either kidney’s structure, abnormalities detected by imaging/histology or in kidney function (biomarkers of kidney injury), present for a minimum of 3 months, with clear clinical significance for health [41]. The assessment of estimated GFR was performed with the conventional CKD-EPI formula [42]. Markers of kidney damage included a urine albumin-to-creatinine ratio ≥ 30 mg/g, persistent hematuria, urine sediment abnormalities, electrolyte disorders and/or other alternative biomarkers when relevant.

### 4.3. Native Coronary Multi-Detector Computed Tomography Angiography

Native coronary multi-detector computed tomography angiography was conducted in synchrony with the 12-lead electrocardiogram in the mid/late diastole following conventional recommendations [43].

### 4.4. Determination of Coronary Artery Calcification

Coronary artery calcification was identified as areas of hyperattenuation > 1 mm^2^ with >130 Hounsfield units (HU) or ≥3 adjacent pixels [44]. Based on the Agatston method we used the weighted sum of lesions with a density > 130 HU to identify the area of calcium accumulation: 130–199 HU (factor 1), 200–299 HU (factor 2), 300–399 HU (factor 3) and ≥400 HU (factor 4) [44].

### 4.5. Echocardiography Examination

All enrolled individuals underwent the standard transthoracic B-mode ultrasound examination, which was performed by highly qualified assessors using a GE Healthcare Vivid E95 scanner (General Electric Company, Strandpromenaden 45, 3183 Horten, Norway) in apical 2- and 4-chamber views. The 2018 Guideline of the American Society of Echocardiography [45] was used to evaluate the conventional hemodynamic parameters, including cardiac dimensions, left ventricular (LV) end-diastolic and end-diastolic volumes, left atrial volume index (LAVI), and LV ejection fraction (LVEF), early diastolic blood filling (E), and the mean longitudinal strain ratio (e′). The estimated E/e′ ratio was expressed as the ratio of the E-wave velocity to the averaged medial and lateral e’ velocity. We evaluated the LV global longitudinal strain (GLS) values by 2D speckle-tracking image after acquiring high-quality echocardiographic data during at least three consequent cardiac cycles. The data were stored in the DICOM (Digital Imaging and Communications in Medicine) format for subsequent analysis.

### 4.6. Clinical Data

The initial demographic characteristics, anthropomorphic data, such as weight, body mass index, waist circumference, and clinical variables, including age, sex, current smoking status, were collected. Patients were invited to respond to a series of enquiries in the form of a questionnaire about their clinical history of acute kidney injury/disease, CKD, hypertension, dyslipidemia, diabetes mellitus, peripheral artery disease, cardiovascular risk factors, history of stroke/TIA, myocardial infarction, revascularization, HF, duration and type of atrial fibrillation, and medication use at the time of their enrolment. Hypertension is defined as a systolic blood pressure ≥ 140 mm Hg or a diastolic blood pressure ≥ 80 mm Hg or taking antihypertensive medication [46]. T2DM was detected according to ADA criteria [47]. Dyslipidemia was diagnosed and managed in accordance with the 2019 European Society for Cardiology/European Atherosclerosis Society Guidelines [48]. Heart failure and determination of its phenotypes were provided according to 2021 European Society of Cardiology Guidelines [49]. Recent TIA was defined as a sudden, focal neurologic deficit that lasts for less than 24 h before study entry. Stroke was defined according to the Stroke Council of the American Heart Association/American Stroke Association scientific statement (2013), and included (a) objective evidence of cerebral, spinal cord, or retinal focal ischemic injury in a defined vascular distribution; or (b) clinical evidence of cerebral, spinal cord, or retinal focal ischemic injury based on symptoms persisting ≥ 24 h [50]. Old myocardial infarction was defined according to Fourth Universal Definition of Myocardial Infarction [51].

### 4.7. Blood Sampling and Biomarker Assessment

Fasting blood samples were collected from the peripheral vein in a BD Vacutainer Serum Tube and stored at room temperature for 30 min to clot. After clotting, the samples were centrifuged at 3000 rpm for 15 min. Samples that were hemolyzed were not used for further evaluation. Serum fractions were aliquoted and stored at −70 °C until ELISA analysis.

Conventional hematological and biochemical parameters, including glucose, electrolytes and creatinine levels, and the lipid profile were routinely obtained from fasting blood tests and determined with a Roche P800 analyzer (Basel, Switzerland) without freezing. Concentrations of NT-proBNP, TNF-alpha, sST2, hs-CRP, fetuin-A, Lp(a) and IL-6 were measured with ELISA kits (Elabscience, 14780 Memorial Dr # 105 Houston, TX, USA). Serum levels of intact FGF-23 were measured using ELISA Kit (Biomedica Medizinprodukte GmbH, Divischgasse 4, 1210, Wien, Austria). Levels of adropin were detected using an ELISA kit (Antibodies.com, Kammakargatan, Stockholm, 47 111 24, Sweden). Analyses were performed according to the manufacturers’ instructions and the results were used for further direct measurements. Each sample was analyzed twice, and the average was used for the final evaluation.

### 4.8. Statistical Analysis

Statistical analyses were carried out using SPSS Statistics v.29 (IBM, 1 Orchard Road, Armonk, NY, USA) and Prism v.10 (GraphPad, 2365 Northside Dr., Ste. 560, San Diego, CA, USA) software. Continuous variables were expressed as the mean (M) ± standard deviation (SD) or the median (Me) with interquartile range (IQR), depending on the data distribution that was routinely evaluated with the Anderson–Darling test. Either a paired t-test or Mann–Whitney test, when appropriate, were used for group comparisons of continuous variables. Categorical data were presented as counts (n) and percentages (%), and appropriate differences between parameters were assessed using Fisher’s exact tests for sparse data, Kruskal–Wallis test for ordinal data, or chi-squared test for nominal data. Spearman’s correlation coefficient (r) was utilized for correlations between circulating biomarkers and other parameters. Plausible predictors of coronary artery calcification were identified using univariate logistic regression and backward stepwise multivariate logistic regression. The factors with a significance of *p* < 0.05 in the univariate log regression analysis were further included in the multivariate log regression model. For each factor odds ratio (OR), 95% confidence interval (CI) and Harrell’s concordance index (c-index) were calculated. The adropin reliability was established using Receiver Operating Curve (ROC) analysis, which involved calculating the area under the curve (AUC), its CI, sensitivity (Se), specificity (Sp) and likelihood ratio. The Youden test was then used to estimate the optimal cut-off point for adropin. The incremental predictive ability of the models was compared to a binary prediction method based on the estimation of integrated discrimination indices (IDIs) and net reclassification improvement (NRI). All tests were two-sided, with *p* value < 0.05 considered statistically significant.

## 5. Conclusions

Low levels of circulating adropin significantly predicted a risk of coronary artery calcification in patients in the early stages of CKD. Perhaps, these findings are promising for further clinical validation of the findings. Further study with larger sample sizes is required to investigate whether the implementation of adropin in routine praxis is economically useful and diagnostically beneficial to screen patients with CKD G1–2 at higher risk of ASCVD.

## Figures and Tables

**Table 1:** Basic characteristics of the patients involved in this study.

Variables	Entire Group Patients with Early (G1–2) CKD (*n* = 337)	Patients with Coronary Calcification (*n* = 196)	Patients Without Coronary Calcification (*n* = 141)	*p* Value
Age (years)	65 (54–77)	68 (55–79)	63 (52–74)	0.044
Male (*n* (%))	216 (64.1)	125 (63.8)	91 (64.5)	0.822
BMI (kg/m^2^)	28.4 ± 6.7	29.6 ± 5.9	27.1 ± 4.6	0.710
Waist circumference (cm)	98 ± 5	98 ± 4	97 ± 6	0.810
WHR (units)	0.90 ± 0.2	0.91 ± 0.1	0.88 ± 0.1	0.750
Smoking (*n* (%))	115 (34.1)	71 (36.2)	44 (31.2)	0.870
Dyslipidemia (*n* (%))	283 (84.0)	172 (87.8)	111 (78.7)	0.061
Hypertension (*n* (%))	269 (79.8)	170 (86.7)	99 (70.2)	0.046
Abdominal obesity (*n* (%))	92 (27.3)	55 (28.1)	37 (26.2)	0.687
T2DM (*n* (%))	128 (38.0)	82 (41.8)	46 (32.6)	0.044
LVH (*n* (%))	273 (81.0)	158 (80.6)	115 (81.6)	0.812
HFpEF (*n* (%))	138 (40.9)	79 (40.3)	59 (41.8)	0.790
Systolic BP (mm Hg)	142 ± 10	143 ± 9	138 ± 7	0.660
Diastolic BP (mm Hg)	84 ± 8	86 ± 6	83 ± 5	0.830
LVEDV (mL)	149 (140–161)	150 (140–163)	149 (138–160)	0.810
LVESV (mL)	68 (61–77)	70 (62–79)	67 (60–78)	0.322
LVEF (%)	55 (51–59)	53 (50–57)	55 (51–59)	0.384
LVMMI (g/m^2^)	142 ± 19	142 ± 16	140 ± 15	0.622
LAVI (mL/m^2^)	34 (31–38)	35 (30–39)	33 (30–37)	0.646
E/e′ (units)	13 ± 6	13 ± 4	12 ± 5	0.716
GLS (%)	−14.5 (−11.6; −17.0)	−14.7 (−11.2; −17.2)	−14.3 (−12.1; −16.7)	0.884
eGFR (mL/min/1.73 m^2^)	78 ± 15	75 ± 13	80 ± 14	0.776
UACR (mg/g)	49 (33–217)	52 (37–226)	46 (32–211)	0.644
Fasting glucose (mmol/L)	4.81 ± 1.24	5.22 ± 1.25	4.67 ± 1.30	0.292
Creatinine (µmol/L)	166 ± 39.1	173 ± 27	159 ± 24	0.655
SUA (mcmol/L)	365 ± 126	370 ± 115	356 ± 119	0.362
Phosphorus (mmol/L)	1.15 ± 0.28	1.15 ± 0.22	1.13 ± 0.20	0.773
Calcium (mmol/L)	2.24 (2.06–2.53)	2.24 (2.10–2.62)	2.22 (2.02–2.50)	0.633
Total cholesterol (mmol/L)	5.70 ± 1.50	5.72 ± 1.42	5.66 ± 1.38	0.551
HDL-C (mmol/L)	0.99 ± 0.17	0.97 ± 0.15	0.99 ± 0.17	0.446
LDL-C (mmol/L)	3.82± 0.21	3.88 ± 0.20	3.79± 0.19	0.515
Triglycerides (mmol/L)	2.21 ± 0.17	2.27 ± 0.16	2.20 ± 0.15	0.524
Lp(a), ng/mL	8.54 (6.32–11.85)	9.77 (6.52–12.40)	8.09 (5.78–11.52)	0.115
sST2 (ng/mL)	9.8 (1.25–16.2)	10.6 (0.77–17.1)	8.5 (1.25–14.6)	0.228
hs-CRP (mg/L)	5.15 (2.23–7.16)	5.21 (2.30–7.30)	5.03 (2.02–6.43)	0.048
TNF-alpha (pg/mL)	2.61 (1.60–3.70)	2.84 (1.92–4.15)	2.32 (1.40–3.53)	0.046
IL-6 (pg/mL)	1.67 (0.54–3.92)	1.74 (0.62–4.15)	1.58 (0.51–3.77)	0.128
NT-proBNP (pmol/mL)	138 (55–219)	142 (53–233)	135 (47–215)	0.563
Adropin (ng/mL)	3.50 (1.90–5.40)	2.85 (1.85–4.07)	3.94 (2.92–5.67)	0.012
Fetuin-A (μg/mL)	54.2 (31.2–72.4)	55.9 (33.6–75.1)	53.8 (30.2–72.5)	0.592
FGF-23 (pg/mL)	93.8 ± 15.2	105.5 ± 13.6	88.2 ± 17.8	0.055
ACEIs (*n* (%))	217 (64.4)	116 (59.2)	101 (71.6)	0.046
Angiotensin-II receptor blockers (*n* (%))	48 (14.2)	37 (18.9)	11 (7.80)	0.026
Beta-blockers (*n* (%))	276 (81.9)	157 (80.1)	119 (84.4)	0.659
Ivabradine (*n* (%))	27 (8.0)	17(8.7)	10 (7.1)	0.769
Calcium channel blockers (*n* (%))	75 (22.3)	37 (18.9)	38 (27.0)	0.040
Loop or thiazide-like diuretics (*n* (%))	161 (47.8)	95 (48.5)	66 (46.8)	0.725
MRA (*n* (%))	95 (28.2)	57 (29.1)	38 (27.0)	0.488
Antiplatelet agents (*n* (%))	87 (25.8)	51 (26.0)	36 (25.5)	0.873
Metformin (*n* (%))	92 (27.3)	58 (30.0)	34 (24.1)	0.554
DPP4 inhibitors (*n* (%))	18 (5.3)	9 (4.6)	9 (6.4)	0.120
GLP-1 receptor agonists (*n* (%))	11 (3.2)	5 (2.6)	6 (4.2)	0.066
SGLT2 inhibitors (*n* (%))	65 (19.3)	39 (19.9)	26 (18.4)	0.854
Statins (*n* (%))	283 (84.0)	172 (87.8)	111 (78.7)	0.061

Abbreviation: BMI, body mass index; BP, blood pressure; CKD, chronic kidney disease; DPP-4, dipeptidyl peptidase-4; eGFR, estimated glomerular filtration rate; E/e′, early diastolic blood filling to longitudinal strain ratio; FGF23, fibroblast growth factor 23; GLS, global longitudinal strain; GLP-1, glucagon-like peptide-1; HFpEF, heart failure with preserved ejection fraction; HDL-C, high-density lipoprotein cholesterol; hs-CRP, high-sensitivity C-reactive protein; IL, interleukin; LAVI, left atrial volume index; LDL-C, low-density lipoprotein cholesterol; LVH, left ventricular hypertrophy; LVEDV, left ventricular end-diastolic volume; LVESV, left ventricular end-systolic volume; LVEF, left ventricular ejection fraction; LVMMI, left ventricle myocardial mass index; MRA, mineralocorticoid receptor antagonists; NT-proBNP, N-terminal natriuretic pro-peptide; SGLT2, sodium–glucose cotransporter-2; SUA, serum uric acid; TNF-alpha, tumor necrosis factor-alpha; T2DM, type 2 diabetes mellitus; UACR, urinary albumin/creatinine ratio; WHR, waist-to-hip ratio.

**Table 2:** Spearman’s correlations coefficients between the levels of circulating biomarkers and other parameters in CKD G1–2 patients with asymptomatic coronary artery calcification.

Variables	Adropin		hs-CRP		TNF-Alpha	
	r	*p*	r	*p*	r	*p*
Age (years)	−0.21	0.024	0.16	0.142	0.12	0.180
BMI (kg/m^2^)	−0.23	0.001	0.19	0.043	0.18	0.050
Systolic BP (mm Hg)	−0.25	0.001	0.08	0.431	0.12	0.220
Diastolic BP (mm Hg)	−0.24	0.001	0.10	0.422	0.12	0.210
LVEF (%)	0.26	0.001	−0.14	0.313	−0.19	0.050
LVMMI (g/m^2^)	−0.31	0.001	−0.21	0.026	−0.13	0.110
LAVI (mL/m^2^)	−0.26	0.016	0.18	0.172	0.14	0.450
GLS (%)	0.32	0.001	−0.17	0.406	−0.13	0.601
Agatston density range	0.42	0.001	−0.20	0.070	−0.21	0.051
eGFR (mL/min/1.73 m^2^)	0.11	0.262	−0.09	0.622	−0.11	0.472
UACR (mg/g)	−0.21	0.012	0.13	0.473	0.19	0.121
Fasting glucose (mmol/L)	−0.19	0.050	0.07	0.542	0.08	0.493
Total cholesterol (mmol/L)	−0.25	0.032	−0.08	0.571	−0.10	0.552
LDL-C (mmol/L)	−0.22	0.040	0.11	0.293	0.13	0.431

Abbreviations: BP, blood pressure; eGFR, estimated glomerular filtration rate; hs-CRP, high-sensitivity C-reactive protein; GLS, global longitudinal strain; LAVI, left atrial volume index; LVEF, left ventricular ejection fraction; LVMMI, left ventricle myocardial mass index; LDL-C, low-density lipoprotein cholesterol; TNF-alpha, tumor necrosis factor-alpha; UACR, urinary albumin/creatinine ratio.

**Table 3:** Predictors of asymptomatic coronary calcification: the results of univariate and multivariate logistic regressions.

Variables	Dependent Variable: Asymptomatic Coronary Calcification							
	Univariate Logistic Regression				Multivariate Logistic Regression			
	OR	95% CI	*p*-Value	C-Index	OR	95% CI	*p*-Value	C-Index
Low adropin vs. elevated adropin	1.26	1.08–1.52	0.001	0.66	1.27	1.13–1.40	0.001	0.01
UACR ≥ 49 mg/g vs. UACR < 49 mg/g	1.02	0.97–1.08	0.438	0.09	-			
hs-CRP ≥ 5.15 mg/L vs. hs-CRP < 5.15 mg/L	1.06	1.01–1.18	0.052	0.12	1.03	1.00–1.10	0.182	0.14
TNF-α ≥ 2.61 pg/mL vs. TNF-α < 2.61 pg/mL	1.09	1.02–1.23	0.048	0.19	1.05	1.00–1.18	0.068	0.13
Hypertension vs. non-hypertension	1.09	1.03–1.22	0.044	0.32	1.09	1.07–1.23	0.042	0.36
T2DM vs. non-T2DM	1.07	1.02–1.15	0.042	0.31	1.05	1.01–1.10	0.044	0.31
LVH vs. non-LVH	1.08	0.96–1.25	0.672	0.11	-			
HFpEF vs. non-HFpEF	1.11	1.02–1.24	0.046	0.37	1.14	1.00–1.28	0.422	0.13
Administration of CCB	0.89	0.71–0.99	0.042	0.39	0.90	0.70–1.02	0.068	0.22
Administration of SGLT2i	0.90	0.82–0.98	0.040	0.42	0.91	0.78–1.00	0.062	0.28

Abbreviations: OR, odds ratio; CCB, calcium channel blockers; CI, confidence interval; LVH, left ventricular hypertrophy; SGLT2i, sodium–glucose cotransporter-2 inhibitor.

**Table 4:** Comparison of predictive models for asymptomatic coronary calcification.

Predictive Models	Dependent Variable: Asymptomatic Coronary Calcification								
	AUC			NRI			IDI		
	M	95% CI	*p* Value	M	95% CI	*p* Value	M	95% CI	*p* Value
Model 1	0.886	0.814–0.957	-	Reference			Reference		
Model 2	0.724	0.699–0.751	0.001	0.09	0.05–0.15	0.688	0.11	0.08–0.16	0.426
Model 3 (T2DM)	0.706	0.625–0.784	0.001	0.07	0.03–0.09	0.772	0.10	0.06–0.17	0.455

Note: *p* value indicates a significant difference compared to Model 1. Model 1: low levels of adropin (<2.95 ng/mL); Model 2: a presence of hypertension; Model 3: a presence of T2DM; Abbreviations: AUC, area under curve; CI, confidence interval; IDI, integrated discrimination indices; M, mean value; NRI, net reclassification improvement; T2DM, type 2 diabetes mellitus.

## Data Availability

The data presented in this study are available on request from the corresponding author due to privacy restrictions.

## Abbreviations

The following abbreviations are used in this manuscript:

AUC	area under curve
BMI	body mass index
BP	blood pressure
CAD	coronary artery disease
CKD	chronic kidney disease
CV	cardiovascular
DPP-4	dipeptidyl peptidase-4
eGFR	estimated glomerular filtration rate
FGF-23	fibroblast growth factor 23
GLP-1	glucagon-like peptide-1
GLS	global longitudinal strain
HDL-C	high-density lipoprotein cholesterol
HFpEF	heart failure with preserved ejection fraction
hs-CRP	high-sensitivity C-reactive protein
HU	Hounsfield units
IL	interleukin
LAVI	left atrial volume index
LDL-C	low-density lipoprotein cholesterol
LVEDV	left ventricular end-diastolic volume
LVEF	left ventricular ejection fraction
LVESV	left ventricular end-systolic volume
LVH	left ventricular hypertrophy
LVMMI	left ventricle myocardial mass index
MRA	mineralocorticoid receptor antagonists
NT-proBNP	N-terminal natriuretic pro-peptide
PI3K	phosphatidylinositol 3-kinase
ROC	Receiver Operating Curve
SGLT2	sodium–glucose cotransporter-2
SUA	serum uric acid
T2DM	type 2 diabetes mellitus
TNF-alpha	tumor necrosis factor-alpha
UACR	urinary albumin/creatinine ratio
WHR	waist-to-hip ratio
